# Correlation of Native Liver Parenchyma T1 and T2 Relaxation Times and Liver Synthetic Function Tests: A Pilot Study

**DOI:** 10.3390/diagnostics11061125

**Published:** 2021-06-20

**Authors:** Ute Lina Fahlenkamp, Jan Kunkel, Katharina Ziegeler, Konrad Neumann, Lisa Christine Adams, Günther Engel, Sarah Maria Böker, Marcus Richard Makowski

**Affiliations:** 1 Department of Radiology, Charité—Universitätsmedizin Berlin, 10117 Berlin, Germany; katharina.ziegeler@charite.de (K.Z.); lisa.adams@charite.de (L.C.A.); guenther.engel@charite.de (G.E.); sarah-maria.boeker@charite.de (S.M.B.); marcus.makowski@tum.de (M.R.M.); 2 Department of Internal Medicine II, Helios Klinikum Emil von Behring, 14165 Berlin, Germany; jan.kunkel@helios-gesundheit.de; 3 Institute for Biometry and Clinical Epidemiology, Charité—Universitätsmedizin Berlin, 10117 Berlin, Germany; konrad.neumann@charite.de; 4 Department of Radiology, Klinikum rechts der Isar der TU München, 81675 Munich, Germany

**Keywords:** MR relaxometry, liver function, mapping, functional imaging

## Abstract

MR relaxometry increasingly contributes to liver imaging. Studies on native relaxation times mainly describe relation to the presence of fibrosis. The hypothesis was that relaxation times are also influenced by other inherent factors, including changes in liver synthesis function. With the approval of the local ethics committee and written informed consent, data from 94 patients referred for liver MR imaging, of which 20 patients had cirrhosis, were included. Additionally to standard sequences, both native T1 and T2 parametric maps and T1 maps in the hepatobiliary phase of gadoxetate disodium were acquired. Associations with laboratory variables were assessed. Altogether, there was a negative correlation between albumin and all acquired relaxation times in cirrhotic patients. In non-cirrhotic patients, only T1 values exhibited a negative correlation with albumin. In all patients, bilirubin correlated significantly with post-contrast T1 relaxation times, whereas native relaxation times correlated only in cirrhotic patients. Evaluating patients with pathological INR values, post-contrast relaxation times were significantly higher, whereas native relaxation times did not correlate. In conclusion, apart from confirming the value of hepatobiliary phase T1 mapping, our results show a correlation of native T1 with serum albumin even in non-cirrhotic liver parenchyma, suggesting a direct influence of liver’s synthesis capacity on T1 relaxation times.

## 1. Introduction

Extensively studied and successfully applied in the field of cardiac diseases, MR relaxometry shows a growing contribution to liver imaging: T1 mapping combined with the liver-specific contrast agent gadoxetate disodium was proven useful to stage liver function in patients, as hepatocellular uptake of gadoxetate disodium into liver cells correlates with clinical liver function tests [1,2,3,4]. Native T1 relaxation times of the liver have, so far, not been studied extensively, and studies are limited to either patients with liver fibrosis or classifications according to the Child–Pugh score [1,5,6,7,8]. Equally, up to now, only very few and mainly preclinical studies evaluating T2 values have been published regarding the liver [9,10], with even fewer clinical approaches [11].

As many pathologies leading to a decreased liver function also result in altered T1 and T2 signal of the liver, a direct comparison of the native relaxation times to liver function is of interest. In this context, it is especially relevant to test to what extent relaxation times may already be influenced by liver function prior to the appearance of cirrhosis, as classified by Child–Pugh.

There are several ways to evaluate liver function, ranging from the simple assessment of serum albumin and prothrombin time, as the hepatic synthesis of albumin decreases in end-stage liver disease, and an increase in prothrombin time depends on the decreased synthesis of liver-derived coagulation factors, over scoring systems derived from those values, such as the above-mentioned Child–Pugh score or the Model of End-Stage Liver Disease [MELD] scores, to more sophisticated tests such as the indocyanine green (ICG) test [12], or the LiMAx test [13] of which the latter are restricted to special indications, e.g., in the preoperative setting, and the clinical scores are limited to higher stages of hepatic disease.

The hypothesis was that irrespective of the presence of irreversible histological changes such as fibrosis, liver relaxation times are influenced by a lot of inherent factors, including changes in liver synthesis function. Therefore, the aim of this study was to see whether relaxation times of liver parenchyma correlate to the serum marker of liver synthesis albumin in unselected adult patients referred for liver MR imaging as part of their routine care. A secondary endpoint was to see to what extent the other laboratory parameters constituting the Child–Pugh score for assessment of liver cirrhosis (international normalized ratio, INR, and bilirubin) correlate to liver parenchyma relaxation times.

## 2. Materials and Methods

### 2.1. Study Population

The study was prospectively approved by and registered with the local ethics committee (Ethikkommission der Charité, EA1/334/16), and the methods were carried out in accordance with the relevant guidelines and regulations. Written informed consent was obtained from all participants.

From December 2016 to June 2019, patients referred for MR examination of the liver with gadoxetate disodium were screened for eligibility. Inclusion criteria were the legal age of majority (18 years and older) and a physical constitution allowing to undergo the full MR examination protocol. Exclusion criteria were pregnancy, metallic implants or functional devices not eligible for MR examination, claustrophobia, a history of allergic reaction to Gd-EOB-DTPA, and a glomerular filtration rate below 30 mL/min.

In total, 94 patients (47 males and 47 females, age range 19–80 years, 56.3 ± 14.8 years, mean ± SD) were included in the study. Indications for referral to liver MRI were exclusion or assessment of metastases in patients with extrahepatic tumors (*n* = 57), exclusion or assessment of hepatocellular carcinoma in patients with cirrhosis (*n* = 20), tumor assessment in patients with suspected cholangiocellular carcinoma (*n* = 6), and other (*n* = 11). In patients with cirrhosis, the diagnosis of cirrhosis had been made beforehand in clinical routine based on physical examination, ancillary testing (e.g., ultrasonography, transient elastography), and/or laboratory analyses, depending on individual requirements.

Details are given in Table 1.

### 2.2. Imaging Protocol

Images were acquired on a clinical 1.5 T MR scanner (Avanto; Siemens Healthineers, Erlangen, Germany) with a 16-channel body-phased array coil. All included patients underwent standard liver MR imaging using the hepatocyte-specific contrast agent gadoxetate disodium (Gd-EOB-DTPA, Primovist^®^, Bayer Vital GmbH), which included an axial T1-weighted spin echo sequence, an axial fat-saturated T2-weighted turbo spin echo sequence acquired with a 2D navigator for abdominal imaging (2D Prospective Acquisition Correction, PACE), an axial T1-weighted dual echo sequence, and axial T1 VIBE (volume-interpolated breath-hold) sequences for dynamic imaging before and 15, 55 s and 2, 5, 10 and 20 min after contrast agent administration and a coronally orientated T1 VIBE sequence for the hepatobiliary phase at least 20 min after contrast agent administration [14].

### 2.3. Study Sequences

Apart from the clinical routine image protocol, patients received balanced steady-state precession readout single-shot Modified Look-Locker Inversion recovery (MOLLI) sequences in the axial plane in three different slice positions before and 20 min after contrast agent administration and, equally in three different slice positions, balanced steady-state free precession T2 mapping sequences (TrueFISP) in the axial plane before contrast-agent administration.

T1 as well as T2 maps were calculated automatically on the scanner console, based on a pixel-by-pixel basis, and displayed by a 12-bit lookup table with a visible color map, with the signal intensity of each of the pixels reflecting their absolute T1 and T2 value. In the case of the TrueFISP T2 mapping sequence, the T2 weighting mainly results from the T2 preparation with variable echo time, e.g., 0 ms (no T2 preparation), 28 ms, and 55 ms. Thus, the T2 contrast is saved, after the T2 preparation, in the Mz magnetization direction. With TrueFISP, only the T2 weighted Mz magnetization is read out. At 1.5T the T2 values of the T2preparated SSFP are very similar to the MESE reference sequence [15].

The imaging parameters for the mapping sequences are shown in Table 2.

### 2.4. Image Analysis

#### 2.4.1. T1 and T2 Relaxometry

All imaging sequences were analyzed on high-resolution dedicated imaging workstations (Centricity PACS, Radiology RA1000, General Electrics).

**Figure 1:**
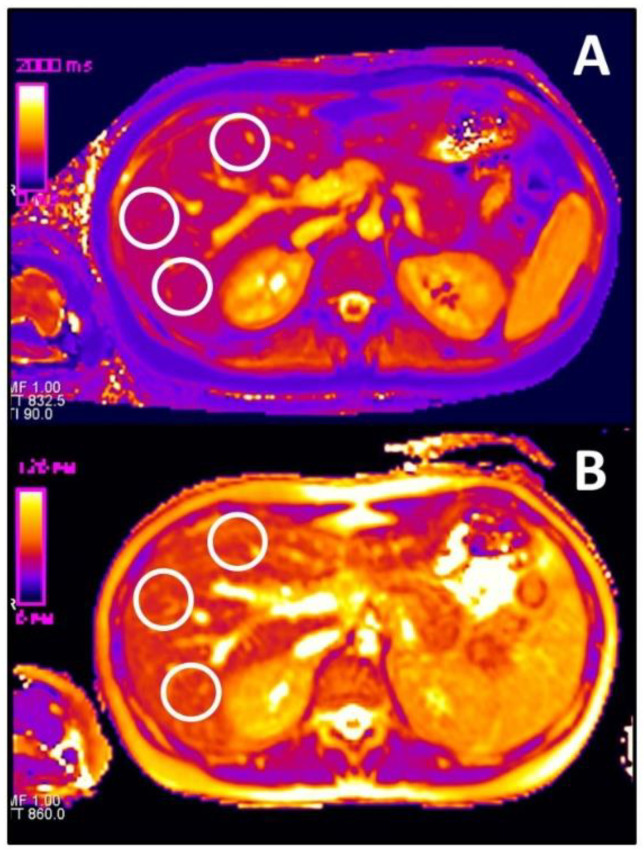
Example of determination of liver parenchyma T1 and T2 relaxation time in a 30-year-old female patient with intrahepatic cholangiolithiasis (not shown). (**A**) represents the T1 map with three circular ROIs, and (**B**) shows the T2 map. The color coding reference bar is shown in both cases on the left of the picture.

**Figure 2:**
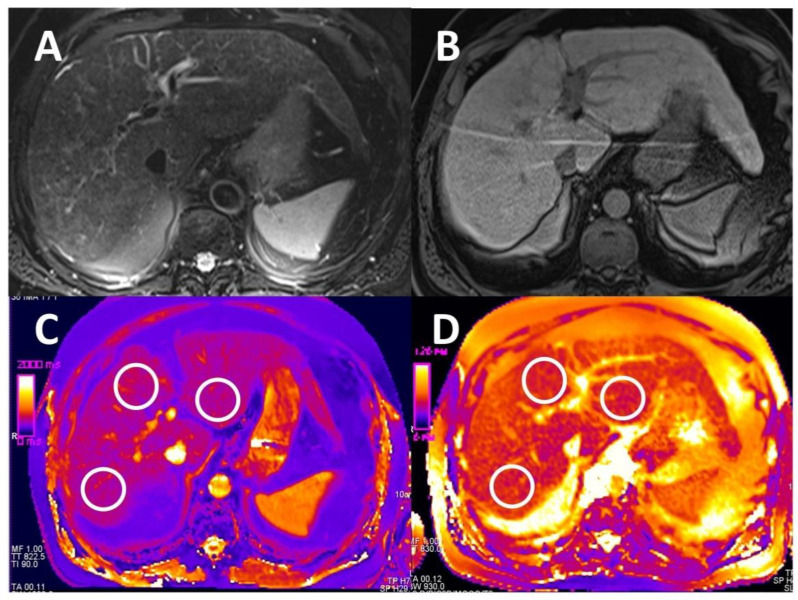
Images of a 65-year-old patient with known cirrhosis in alcoholic liver disease. (**A**) is a T2 weighted, fat-saturated image (TR/TE: 4043/79 ms), and (**B**) a T1 GRE sequence (TR/TE: 4.74/2.38 ms) 20 min after application of a hepatocyte-specific contrast agent. (**C**) shows the T1 map, and (**D**) the T2 map, both with an ROI placed to measure mean relaxation time.

On T1 and T2 maps as well as on T1 VIBE sequences acquired in the hepatobiliary phase 20 min after application of gadoxetate disodium, three circular ROI chosen as large as possible were placed in the liver parenchyma taking care to avoid focal alterations and lesions, as well as larger vessels. The ROI was adjusted anew to every single of the three different slice positions and the different mapping sequences. Image examples are given in Figure 1 and Figure 2. For the final analysis, mean values of the three ROIs on the different slice positions were calculated. The reviewing radiologist was blinded to all laboratory data as well as all patient-specific information.

A subgroup analysis to evaluate the robustness of T1 and T2 measurements was performed in *n* = 10 patients, collecting the size of the ROI as well as minimal, maximal, and mean relaxation time.

#### 2.4.2. Laboratory Values

Acquisition of laboratory data was not part of the prospectively planned study, and thus, data had to be collected retrospectively. Therefore, the electronic medical records were searched for documented pertinent laboratory data albumin, INR, and bilirubin. The patients’ data were excluded from the final statistical analysis, if the time span between the MR examination and blood sampling was longer than 35 days.

#### 2.4.3. Statistical Analysis

Native T1 and T2 times, as well as T1 times in the hepatobiliary phase (T1HBP), are given in minimum, maximum, means, and standard deviation, as well as total bilirubin, albumin, and INR. Correlation between serum parameters and relaxation times were assessed using Pearson’s r. Correlation coefficients were interpreted according to the publication by Schober et al. [16], with an r of 0.00–0.09 indicating a negligible correlation, 0.10–0.39 indicating a weak correlation, 0.40–0.60 indicating a moderate correlation, 0.70–0.89 indicating a strong correlation, and 0.90–1.00 indicating a very strong correlation. For INR, because of the U-shaped distribution of pathological values, mean relaxation times were compared between patients with normal (0.8–1.2) values and pathological values. As the study is observational, all *p*-values are exploratory. No adjustment for multiple testing was performed. All statistical analyses were carried out using IBM SPSS statistics version 25.

## 3. Results

Ninety-four patient data sets were evaluated for final eligibility. Of these, suitable laboratory data were available for 60 to 78 patients, depending on the parameter. A comprehensive summary of laboratory findings, as well as T1 and T2 values, are given in Table 3. Of the 65 patients with available INR values, 19 (29.2%) had pathological values of >1.2. None of the patients had an INR of below 0.8.

Within the subgroup analysis to evaluate the robustness of T1 and T2 measurements, mean size of the ROIs was 1090.44 mm^2^. The standard deviation within ROIs was 143.30 ms for T1, and 12.43 ms for T2. Intrasubject standard deviation of the different ROIs on one map was on average 22.22 ms for T1, and 3.56 ms for T2.

### 3.1. Correlation Analyses

All correlation analyses are summarized in Table 4. Significant values are given in bold. Pearson’s correlations were performed separately for patients with cirrhosis and those with other pathologies, and subgroup analyses were performed for patients with laboratory data from within a time span of 10 days or less.

#### 3.1.1. Albumin

Albumin values were available in 60 patients, 33 of them within 10 days of the MRI examination. Altogether, there was a strong to very strong negative correlation between albumin and native T1 and T2, as well as post contrast T1 relaxation times in cirrhotic patients. This correlation was much weaker in non-cirrhotic patients, where only native and post-contrast T1 values exhibited a weak to moderate negative correlation with albumin.

#### 3.1.2. Bilirubin

Bilirubin values were available 78 patients, of which 39 were from within ten days of the MRI. In both cirrhotic and non-cirrhotic patients, bilirubin correlated significantly with post-contrast T1 relaxation times—the correlation was moderate to strong in cirrhotic patients and moderate in non-cirrhotic patients. Furthermore, both native T2 and T1 values correlated significantly (moderately to strongly) with bilirubin in cirrhotic patients.

#### 3.1.3. INR

INR values were available for 65 patients, 39 of which were collected within 10 days of the MRI exam. Post-contrast relaxation times were significantly higher in all patients with pathological INR values. Native relaxation times did not differ significantly between groups. Correlations are given in Table 5, and significant values are written in bold. 

## 4. Discussion

The liver is a complex organ with multiple roles in metabolism. In addition to carbohydrate, protein, and fat metabolism, as well as detoxification, it synthesizes plasma proteins, including clotting factors. Therefore, in clinical practice, serum albumin, and prothrombin time (with its derived measure INR) are often considered as tests of liver function. As such, they are part of the Child–Pugh score used in clinical practice to assess the severity of chronic liver disease [17].

The results of this study show that T1 relaxation times of liver parenchyma correlate to the serum marker of liver synthesis albumin in both cirrhotic parenchymal alteration and in patients with normal liver structure. In non-cirrhotic patients, this correlation was even more pronounced than the established marker of gadoxetate disodium uptake in the hepatobiliary phase making native T1 relaxation times a valuable tool for advanced liver imaging. Concerning T2 relaxation times, a significant correlation was only present in patients with cirrhosis. To the other two laboratory markers contributing to the Child–Pugh score, T1 and T2 relaxation times only show a much lower, if even, correlation.

The relaxation times are tissue parameters that are dependent on the physical, chemical, and biological characteristics of the tissue examined [1]. Studies concerning native mapping in liver imaging have shown that T1-relaxation time increases with fibrosis, as measured on histopathology [6,7,8]. Other studies have shown that T1 was able to discriminate between healthy subjects and those with chronic liver disease as classified by the Child–Pugh score [1,3], which goes along with the evidence that clinical Child–Pugh scores are related to the histopathological fibrosis stage [18,19]. Native T2 relaxation times are less well investigated, but, apparently, T2 relaxation times increase with every Child–Pugh class, even though not in a statistically significant way [1].

Still, taking into account the liver’s manifold and complex roles, one may assume that there are other aspects influencing relaxation time, that there may even be a measurable influence in subjects whose liver function is normal.

We chose serum albumin as a parameter of liver synthetic function, as its measurement is not restricted to patients with clinically suspected or known impaired liver function, and therefore, available in a broad spectrum of patients. The comparison to INR and bilirubin was performed as they are the two other laboratory parameters contributing to the Child–Pugh score of chronic liver disease [17].

Our results that T1 relaxation times of liver parenchyma correlate to the serum marker of liver synthesis albumin in both cirrhotic and non-cirrhotic patients and—in a selected subgroup—even better compared to the surrogate marker of gadoxetate disodium uptake in the hepatobiliary phase took us to the conclusion, that fibrosis is not the only important factor leading to altered relaxation times in patients with impaired liver function. Apparently, relaxation times already show a tendency in patients without impaired liver function depending on the level of serum albumin. As albumin constitutes about 55% of the total plasma protein, and the liver is responsible for 85–90% of circulating protein volume, a direct effect of the turnover of proteins in the liver on relaxation time seems probable [20].

With T2 relaxation times, however, the correlation was much lower and was only present in patients with cirrhosis, which goes along with the findings of another study evaluating T2 relaxation times in liver disease [11], which were related to a certain inflammation in liver fibrosis.

There was a measurable correlation of gadoxetate disodium uptake and serum bilirubin levels, which has been shown in preceding studies, and which can be related to the fact that both unconjugated bilirubin and gadoxetate disodium are taken up into hepatocytes by the OATP1B1 transporter [21,22,23]. Effects on native relaxation times, however, are not present in a significant way with bilirubin, if present at all.

With INR, whereas post-contrast relaxation times were significantly higher in all patients with pathologically altered INR values, native relaxation times did not differ significantly between groups. Apparently, even though clotting factors can also be regarded as markers for liver synthesis function, their impact, including storage in the liver, does not seem to be high enough to produce an effect analogous to albumin on T1 relaxation times.

Of course, some confounding aspects have to be discussed: It is well known that even though regarded as tests for liver synthesis function in clinical practice, neither of the tests is specific for liver disease: Albumin serum levels are also reduced in patients with malnutrition or malabsorption, protein-losing enteropathy, or nephrotic syndrome, and prothrombin time is increased with cumarin treatment or vitamin K deficiency. For our study cohort, even though we cannot confidently exclude malnutrition as a confounding factor, according to the accessible medical records, the other typical preconditions for serum albumin alterations were not present in our study population, and none of the patients was on cumarin treatment.

The study has certain limitations. The number of patients with less than 10 days between blood sample and examination is small, and the number of patients with a blood sample taken on the same day is even lower. Therefore, especially our results concerning INR have to be named preliminary. With serum albumin, one may assume confident contribution of data within a longer time span, as it does not usually undergo short-term fluctuations. Secondly, ROI placement may succumb to a certain bias of selection, as not the entire functioning liver parenchyma could be incorporated into measurements, yet, we attempted to receive a reliable value by averaging measurements of three different slice positions.

## 5. Conclusions

In conclusion, apart from confirming the value of hepatobiliary phase T1 mapping, we could demonstrate in this study that native T1 values of liver parenchyma, but not native T2 values, correlate with serum albumin even in patients without cirrhotic transformation. This suggests that T1 relaxation times of the liver may also to a relevant part influenced by liver synthesis capacity.

## Figures and Tables

**Table 1:** Indications for referral to liver MRI.

**Entire Study Cohort**		*n* = 94	
Referral to MRI due to…			
**Extrahepatic tumor**		*n* = 57	
of which			
	Breast		*n* = 22
	Neuroendocrine		*n* = 10
	Thyroid gland		*n* = 1
	Colorectal		*n* = 9
	Lung		*n* = 1
	Skin		*n* = 6
	Cervix uteri		*n* = 1
	Prostate		*n* = 1
	Stomach		*n* = 1
	Kidney		*n* = 2
	Testes		*n* = 2
	Pancreas		*n* = 1
**Cirrhosis**		*n* = 20	
**Cholangiocellular carcinoma**		*n* = 6	
**Diverse indications**		*n* = 11	
of which			
	PSC		*n* = 3
	Liver lesion of unknown entity		*n* = 5
	Choledocholithiasis, suspected underlying malignancy		*n* = 2
	Liver abscess, suspected underlying malignancy		*n* = 1

MRI magnetic resonance imaging; PSC Primary sclerosing cholangitis.

**Table 2:** Sequence parameters of the T1 and T2 mapping sequences. Parameters for T1 mapping apply for both precontrast and hepatobiliary phase imaging.

Sequence	T1 Map (MOLLI)	T2 Map (True FISP)
Scan plane	Axial	Axial
Voxel size (mm)	2.4 × 1.6 × 6.0	2.6 × 2.1 × 6.0
Number of slices	3	3
Slice thickness (mm)	6	6
TR/TE (ms)	912/1.08	227.29/1.13
Averages	1	1
FoV (mm)	320	319
Flip angle (°)	35	70
Bandwidth (Hz/Pixel)	1028	930
Fat saturation	None	None
Number of inversions	3	-
MOLLI TI start (ms)	90	-
MOLLI TI increment (ms)	80	-
MOLLI trigger delay (ms)	160	-
Number of T2 preparations	-	3
Echo spacing (ms)	-	2.5

MOLLI: Modified Look-Locker Inversion recovery; FISP: Fast imaging with steady state precession; TR: Repetition time; TE: Echo time; FoV: Field of view; TI: Inversion time.

**Table 3:** Descriptive Results.

Parameter	N	Mean	SD	Lower Limit	Upper Limit
Age	94	56.3	14.8	19	80
Albumin (g/L)	60	40.3	5.5	23.0	48.4
INR	65	1.2	0.4	0.8	3.0
Bilirubin (mg/dL)	78	0.9	1.9	0.2	14.0
T2 value (ms)	94	55.9	6.1	41.7	84.0
T1 value (ms)	94	563.3	74.9	319.7	819.0
T1HBP value (ms)	94	218	73.3	138	460

INR: international normalized ratio; T1HBP: T1 value in the hepatobiliary phase after contrast agent administration.

**Table 4:** Pearson’s correlations for albumin and bilirubin with relaxation times.

Parameter			N	Correlation T2		Correlation T1		Correlation T1HBP	
				r	*p*	r	*p*	r	*p*
Albumin	Cirrhotic	Within 10 days	9	**−0.829**	**0.006**	**−0.918**	**<0.001**	**−0.942**	**<0.001**
		Any time point	18	**−0.585**	**0.011**	**−0.574**	**0.013**	**−0.862**	**<0.001**
	Non- cirrhotic	Within 10 days	24	−0.288	0.172	**−0.437**	**0.033**	−0.319	0.129
		Any time point	42	−0.202	0.199	**−0.412**	**0.007**	**−0.312**	**0.044**
Bilirubin	Cirrhotic	Within 10 days	9	0.563	0.115	**0.840**	**0.005**	**0.808**	**0.008**
		Any time point	19	**0.524**	**0.021**	**0.576**	**0.010**	**0.629**	**0.004**
	Non- cirrhotic	Within 10 days	30	0.157	0.407	0.308	0.098	**0.409**	**0.025**
		Any time point	59	0.084	0.527	0.131	0.324	**0.387**	**0.002**

**Table 5:** Pearson’s correlations for INR with relaxation times.

	INR	N	Mean T2 Value	*p*	Mean T1 Value	*p*	Mean T1HBP Value	*p*
Cirrhotic	Normal	3	54.67	0.167	559.00	0.262	**208.56**	0.048
	Path.	6	63.33		636.78		**353.44**	
Non- cirrhotic	Normal	26	56.04	0.071	556.26	0.536	**204.24**	0.026
	Path.	4	50.17		548.75		**276.00**	

## Data Availability

The data sets used and/or analyzed during the current study are available from the corresponding author on reasonable request.
